# When the brain, but not the person, remembers: Cortical reinstatement is modulated by retrieval goal in developmental amnesia

**DOI:** 10.1016/j.neuropsychologia.2021.107788

**Published:** 2021-04-16

**Authors:** Rachael L. Elward, Michael D. Rugg, Faraneh Vargha-Khadem

**Affiliations:** a UCL Great Ormond Street Institute for Child Health, London, UK; b London South Bank University, London, UK; c Center for Vital Longevity and School of Behavioral and Brain Sciences, University of Texas at Dallas, USA; d School of Psychology, University of East Anglia, UK

**Keywords:** fMRI, Recollection, Strategic-retrieval, Scene reinstatement, Memory retrieval

## Abstract

Developmental amnesia (DA) is associated with early hippocampal damage and subsequent episodic amnesia emerging in childhood alongside age-appropriate development of semantic knowledge. We employed fMRI to assess whether patients with DA show evidence of ‘cortical reinstatement’, a neural correlate of episodic memory, despite their amnesia. At study, 23 participants (5 patients) were presented with words overlaid on a scene or a scrambled image for later recognition. Scene reinstatement was indexed by scene memory effects (greater activity for previously presented words paired with a scene rather than scrambled images) that overlapped with scene perception effects. Patients with DA demonstrated scene reinstatement effects in the parahippocampal and retrosplenial cortex that were equivalent to those shown by healthy controls. Behaviourally, however, patients with DA showed markedly impaired scene memory. The data indicate that reinstatement can occur despite hippocampal damage, but that cortical reinstatement is insufficient to support accurate memory performance. Furthermore, scene reinstatement effects were diminished during a retrieval task in which scene information was not relevant for accurate responding, indicating that strategic mnemonic processes operate normally in DA. The data suggest that cortical reinstatement of trial-specific contextual information is decoupled from the experience of recollection in the presence of severe hippocampal atrophy.

## Highlights

• Patients show fMRI reinstatement effects despite hippocampal pathology and episodic amnesia.
• Cortical reinstatement may reflect an implicit memory process.
• Reinstatement indicates that implicit pattern completion processes operate in DA.
• DA Patients engage in strategic retrieval gating of task-irrelevant information.
• Reinstatement may enable cortical learning, irrespective of explicit memory.

## 1 Introduction

Declarative memory (including semantic and episodic memory) develops over the course of childhood (for reviews see Bauer, 2013; Mullally and Maguire, 2014). Infants acquire a vast amount of semantic information (including conceptual knowledge and vocabulary) in the first years of life. Episodic-like memories (e.g. imitating actions after a delay) can be acquired in infancy but are more rapidly forgotten than in later childhood or adulthood (Bauer, 2015). Episodic memories for events in one's life emerge in middle childhood (between 3 and 7 years of age) (Bauer et al., 2007; Ghetti and Lee, 2011), marking the beginning of a personal autobiography (Nelson, 1992). Adults are able to mentally travel back in time to specific moments of their childhood and relive past events as a personal memory in autonoetic consciousness (Tulving, 1983). The ontogeny of episodic memory occurs in parallel with the protracted structural and functional development of the brain systems that support episodic memory, in particular, the hippocampal formation (Bachevalier and Vargha-Khadem, 2005; Hunsaker et al., 2014). It is likely that episodic memory abilities emerge from this development. Indeed, if the neural systems that support memory fail to develop episodic memory is irrevocably impaired, leading to Developmental Amnesia (DA). This memory disorder emerges after bilateral hippocampal damage in early life (Brizzolara et al., 2003; Vargha-Khadem, 1997). A remarkable feature of DA is the dissociation between semantic memory and episodic memory, whereby the former continues to be accrued throughout the developmental trajectory, while the latter remains chronically impaired. Children with DA learn language at age-appropriate levels, and acquire a massive amount of semantic knowledge over their lifespan, but they cannot recall past events of their lives (Baddeley et al., 2001; Elward and Vargha-Khadem, 2018; Gardiner et al., 2008; Jonin et al., 2018).

Like typically-developing infants and young children, patients with selective, bilateral hippocampal damage learn semantic information well before this structure has matured, and before episodic memory function has emerged. This early semantic learning is held not to involve autonoesis or the subjective experience of self in time (Tulving, 2002) and, based on an anatomo-functional model of cognitive memory, is likely to proceed via the perirhinal and entorhinal cortices, independently of the hippocampus (Mishkin et al., 1997). However, there is considerable debate as to the extent of a division of labor, or a reciprocal interaction, between the cortical versus the hippocampal components of the medial temporal lobe serving episodic and semantic memory (for recent reviews see Duff et al., 2020; Renoult et al., 2019; Moscovitch et al., 2016). It should be noted, however, that models accounting for the extent of hippocampal involvement in semantic memory and episodic retrieval (e.g. Covington et al., 2018; Manns et al., 2003) are based on data from patients who had developed normal episodic and semantic memory prior to their adult-onset hippocampal injury. As such, these models do not address the puzzle of how patients with DA acquire semantic world knowledge given that their hippocampal damage has occurred before any memory function has developed. Specifically, if hippocampal integrity is crucial for learning, then how do patients with DA acquire language and semantic knowledge about the world? One way to address this question is to investigate the ways in which the hippocampus supports episodic memory, and to consider whether patients with DA are able to engage some episodic memory-related processes in a manner sufficient to support semantic learning irrespective of their subjective experience of remembering (autonoetic consciousness).

**Fig. 1:**
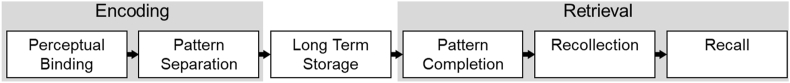
Episodic memory related processes that are associated with the hippocampal circuitry. Patients with DA have difficulty with recall.

Although its specific role in episodic memory is hotly debated, several cognitive processes have been associated with hippocampal function (see Hannula and Duff, 2017; for review). These processes include (1) the high-resolution “binding” of perceptual elements at encoding to form a unique episodic representation (c.f., Ekstrom and Yonelinas, 2020), (2) the “pattern separation” of this episodic representation so that it can be stored independently of representations of similar events, (3) long-term storage of the representation (4) “pattern completion”, whereby a partial cue (e.g. the word “concert”), can be sufficient to prompt the retrieval of the entire memory (e.g. the sights, sounds and feelings of attending a particular musical concert) - a process linked to the reinstatement of the mnemonic representation in the cortex, (5) the subjective experience of “recollection”, whereby a prior event may be subjectively re-experienced (“mental time travel”), and (6) memory-guided behaviour, including recall, which enables us to tell anecdotes about our life events. Patients with DA have marked difficulty with episodic recall, but it is less clear which stage of mnemonic processing, prior to recall, is the point at which episodic memory fails (see Fig. 1).

There is growing evidence that patients with DA are able to accomplish some aspects of episodic memory-related processing, perhaps by relying on remnant hippocampal tissue, or by recruiting extra-hippocampal tissue such as the rhinal cortices. Patients with DA are able to bind information in working memory, but a memory deficit emerges over increasing delays (Baddeley et al., 2010; Allen et al., 2014; Jeneson et al., 2011). There is some evidence, however, that when associative memory is probed with a test of recognition memory, patients with DA are unimpaired relative to controls over study-test delays of up to several minutes (Vargha-Khadem et al., 1997). Buck and colleagues presented an associative cued recall and recognition memory test to five patients with DA. Here, 10 pairs of words were learnt over three consecutive trials, and tested with cued recall after **a 15-min** delay, followed by a multiple-choice associative recognition test. Patients were asked to identify the associated word-pair from a list of three words (one correctly paired word, one familiar foil that was associated with another word-pair, and one novel word). In this study, patients with DA were able to recognise the associated word-pair with 78% accuracy over the delay period but were unable to retrieve the paired-associates using cued recall; 10% accuracy (Buck et al., personal communication, see also Buck et al., 2020). These data indicate that, under some conditions, associations may be encoded and retained in patients with DA and made available for recognition but not for recall.

Why can patients with DA not recall information that has been bound and stored in memory? One possibility is that the patients are able to retrieve partial information about prior events, but that the retrieved information is insufficient to support recall. This possibility is consistent with the ‘Precision and Binding Model’, which states that episodic memory requires high-resolution binding of multiple perceptual elements (Ekstrom and Yonelinas, 2020; Kolarik et al., 2019). According to this model, patients with hippocampal damage form only imprecise memory representations. It is possible therefore that DA patients are able to retrieve perceptual elements of a prior experience (via pattern completion processes), but that the retrieved information lacks the vivid detail of a personal episodic memory and is therefore insufficient to support recall. If so, this may explain how patients with DA are able to acquire semantic information in the presence of episodic amnesia. In order to build a semantic understanding of a concept (e.g. music is performed at concerts) one does not need to recollect the precise details of a single autobiographical experience. That is, partial retrieval of episodic information may be sufficient to support semantic learning in the cortex, in the absence of the experience of recollection. This raises the question, is there any evidence that patients with DA are able to retrieve partial or imprecise information associated with their episodic experiences? Unfortunately, it is difficult to experimentally assess memory retrieval in patients with DA through self-report since all subjectively experienced memories have occurred in the presence of hippocampal damage. Thus, memories that are reported to be “recollective” or “vivid” by patients with DA are likely to be qualitatively different from those of control volunteers (Brandt et al., 2006; Gardiner et al., 2006; Maguire et al., 2001). A more objective method for assessing partial memory retrieval in patients with DA might be to examine cortical reinstatement effects.

Cortical reinstatement is the phenomenon whereby patterns of neural activity elicited during the encoding of an event are recapitulated in the cortex during retrieval (Alvarez and Squire, 1994; McClelland et al., 1995; Danker and Anderson, 2010). Functional imaging studies of healthy adults have reported that stronger cortical reinstatement is associated with more accurate and more confident memory judgements. This suggests that reinstatement may be taken as an objective measure of the amount of episodic information that is retrieved from memory (Gordon et al., 2014; Hofstetter et al., 2012; Huijbers et al., 2011; Kuhl et al., 2012; Liang and Preston, 2017; Slotnick, 2009; Staresina et al., 2012; Thakral et al., 2015). Importantly, however, Thakral et al. (2017) reported that cortical reinstatement can be evidenced in fMRI BOLD signals when participants *fail* to recollect a prior episode. That is, participants do not have the subjective experience that they can remember the prior event, but cortical reinstatement effects are observed nonetheless. This finding suggests that, in at least some circumstances, reinstatement can reflect an implicit episodic memory process in which information from a prior event is reactivated. This process may facilitate the experience of episodic memory in healthy adults, but is not sufficient for the experience to occur (see also Kahn et al., 2004; Gagnon et al., 2018 for similar findings). By examining cortical reinstatement effects in DA, we may be able to *infer* that pattern completion processes can occur at retrieval, regardless of whether there is an accompanying subjective experience of recollection, even in amnesic populations. This would suggest that episodic information can be “retrieved” without the awareness, and thus, may contribute to formation of semantic memory.

In addition to the six processing stages described above, strategic memory processes are also thought to be crucial for accurate memory-driven responses (Halamish et al., 2012; Henson et al, 1999, 2000; Rugg and Wilding, 2000). That is, when presented with a retrieval cue such as “who did you go to the concert with?“, the ensuing memory search needs to be directed toward goal-appropriate information (i.e. people) and directed away from goal-irrelevant information (e.g. music, lights). It has been proposed that the development of mnemonic control processes depends upon mnemonic experience to mature effectively (Fandakova et al, 2018a, 2018b; Luna et al., 2015). Thus, patients with DA may not have had the opportunity to develop the processes that support strategic control of memory retrieval. Cortical reinstatement effects have been utilised to investigate these strategic operations in healthy adults. In a prior study, we reported that healthy adults exercise control over reinstatement effects in a goal-congruent manner (Elward and Rugg, 2015). In that study, (which employed a paradigm similar to that adopted here) the participants demonstrated cortical reinstatement of scene-specific information when task requirements necessitated scene retrieval (was the test word presented at study with an urban or rural scene?). However, scene reinstatement effects were attenuated when scene memory was task irrelevant (was the test word presented at study on the left or right side of the display monitor?). These findings indicate that, in healthy adults, goal-relevant mnemonic details can be selectively reinstated. The investigation of strategic retrieval processing in DA is potentially an important avenue for research. If patients with DA are unable to adopt appropriate strategic retrieval strategies, then they would be expected to show equivalent reinstatement effects irrespective of the retrieval goal.

Through the examination of scene reinstatement effects, here we evaluate 1) whether patients with DA show evidence of retrieval of mnemonic content, despite their poor memory performance, and 2) whether they are capable of engaging goal-dependent retrieval strategies.

## 2 Materials and methods

### 2.1 Participants

All participants provided their informed consent to participate. The research project was approved by the Hampstead NHS Research Ethics Committee and overseen by the Research and Development Department of Great Ormond Street Hospital for Children NHS Foundation Trust, and the UCL Great Ormond Street Institute of Child Health, London, UK.

Nineteen control participants contributed data (11 male, aged 20–42); the data from one participant was excluded due to movement artefact. Control participants were recruited from the Psychology Department of UCL and through flyers and email advertisements. All participants indicated that they were right-handed, spoke English as a first language, had normal or corrected vision, were in good health, had no history of a serious medical, neurological or psychiatric condition, were not born preterm and were not regularly taking medication. Participants were compensated with £30 for participation in the research.

Five adult males with DA also participated (see Table 1). The details of these patients have been previously described (Dzieciol et al., 2017). On the Wechsler Adult Memory Scale (WMS) III, patients showed good working memory, but impaired recall, especially for auditory verbal material. Hippocampal volumes were measured for each patient and showed significantly reduced volume loss bilaterally (30–52%) relative to normal (Dzieciol et al., 2017). Notably, the hippocampal volume measurements of patient DA09 were remeasured in 2020 and this patient's volume reduction was shown to be less extensive than previously recorded. His hippocampus on the whole is reduced in volume by 14% relative to normal. This new measurement was acquired after the patient was recruited and tested for this study and we retained this patient in the analyses here as a DA patient, based on his cognitive memory profile, and his moderate hippocampal volume loss (Guderian et al., 2015).

**Table 1:** Participant characteristics. Hippocampal volume reductions (HVR) are reported as the percentage of the mean volume of a group of controls (Cooper et al., 2015). Standardised scores (x = 100; sd, 15) from Wechsler Adult Memory Scale III are as follows, Aud Del = Auditory Delayed, Vis Del = Visual Delayed, Rec = Recognition, Gen Mem = General Memory, WM = Working Memory.

Participant	HVR	Age	Wechsler Memory Scale III				
			Aud. Del	Vis. Del.	Rec	Gen Mem.	WM
Average Control	–	29	114	103	107	110	123
Average DA	43%	31	56	70	87	62	119
DA 01	50%	38	58*	68*	70	52*	124
DA 09	14%	32	52*	65*	95	62*	96
DA 12	48%	32	52*	75	75	60*	99
DA 15	52%	30	52*	68*	85	60*	141
DA 16	50%	22	67*	75	110	77	136

* Standard Scores <70 on WMS are in the “Extremely Low” range.

### 2.2 Procedures

The memory paradigm was administered as a single study-test cycle. The study phase took place outside of the scanner using a laptop computer and was followed by a short interval (approx. 15 mins). The test phase was completed inside the MRI scanner. Following the test phase, structural T1-weighted MR images were acquired. Finally, a functional localiser was administered to localise scene-selective cortical regions. Each of these procedures is described below.

#### 2.2.1 Study phase

**Fig. 2:**
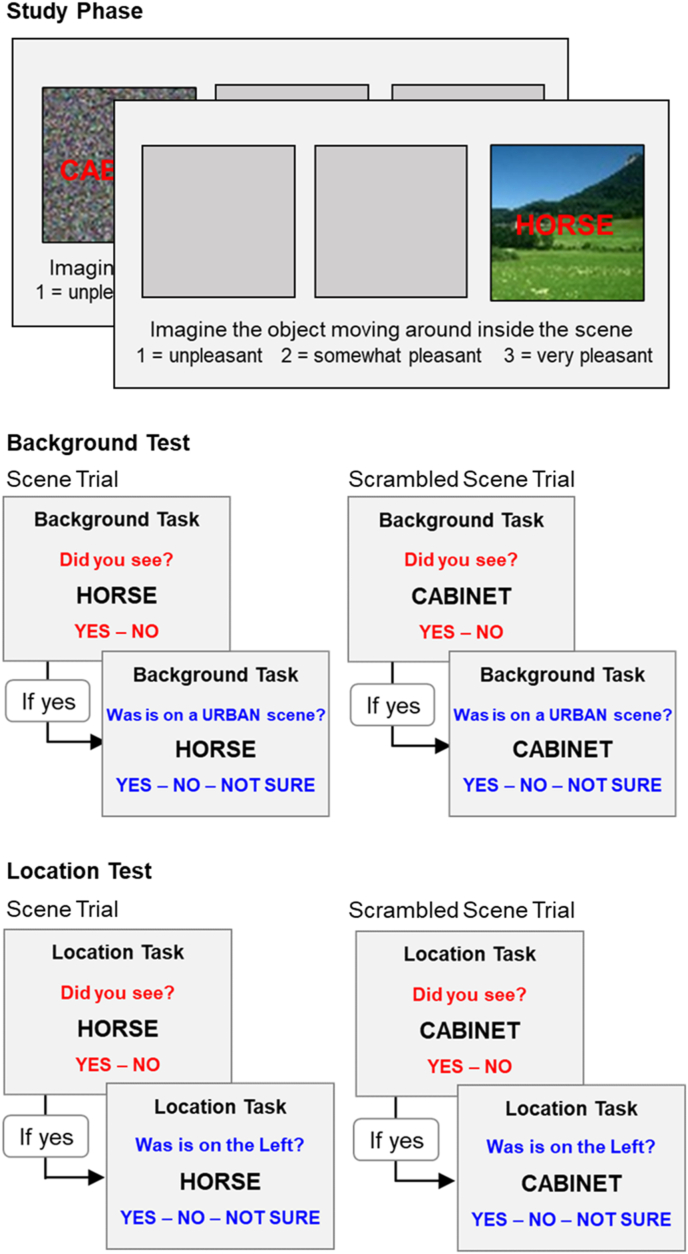
Schematic of the experimental protocol and the key trial types for the fMRI analysis. The study phase was conducted outside of the MRI scanner. The memory test phase was conducted during fMRI data acquisition. In both memory tasks, the test trials began with a recognition question (in red font) and if participants indicated that they recognised the word, proceeded to a context memory question (in blue font). The key trial types are words that had been previously paired with scenes and scrambled scenes in the location and background tests. (For interpretation of the references to colour in this figure legend, the reader is referred to the Web version of this article.)

This phase was similar to that employed in prior work in healthy adults (Elward and Rugg, 2015). A schematic of the study phase is presented in Fig. 2. Throughout the study phase, three squares were presented on the display monitor, one at the centre, one on the left, and one on the right. On each trial, a fixation cross was presented in one of the squares for 200 ms. Then, a word was presented in place of the fixation cross, and simultaneously, the same word was presented auditorily. Two hundred milliseconds after the onset of the word, a scene image was presented in the same location as the word. Each image was trial-unique and belonged to one of three categories: Rural Scene, Urban Scene, or a Scrambled Scene. Scrambled scenes were created by randomly shuffling the pixels within each scene image to create unrecognisable control stimuli. Scenes were selected from the Computational Visual Cognition Laboratory database http://cvcl.mit.edu/database.htm). Participants were instructed to imagine the object denoted by the word moving around inside the scene or moving around inside the scrambled image and then to rate the pleasantness of the resulting image. To ensure that patients did not forget the task instructions, the following text was displayed at the bottom of the display screen throughout the study phase: “Imagine the object moving around inside the scene; ' 1 = unpleasant 2 = somewhat pleasant 3 = very pleasant'“. The image and the word were presented for 5500 ms. For a 400 ms inter-trial-interval, the three grey squares and the task instructions remained on display. The total duration of one trial was 6300 ms. Twenty images in each category (Urban Scene, Rural Scene, Scrambled Scene) were presented in each position (Centre, Left, Right) for a total of 180 study trials. Trials were presented in a pseudorandom order such that no more than three consecutive trials were presented at the same location, or with the same image category. The trials were presented over two blocks each lasting 9.5 min. The break between the blocks was untimed, the second block beginning when the participant indicated that they were ready to continue.

Once the study phase was complete the test instructions were given and a practice test was administered before participants moved to the MRI scanner. While in the MRI scanner, the test instructions and the practice test were repeated.

#### 2.2.2 Test phase

The memory test consisted of two retrieval tasks: the ‘Background’ Task and the ‘Location’ Task. In the Background Task, participants were required to retrieve the background image that accompanied each studied word, and during the location task participants were asked to retrieve the location in which a study word was presented. The tasks were blocked such that participants completed 60 trials of one task before switching to the other. The tasks were presented alternately in an ABAB sequence that comprised a total of 240 test trials (180 words from the encoding phase interspersed with 60 new words). A reminder of the current retrieval task (i.e. Location Task, Background Task) was displayed on the top of the screen throughout each task block. A schematic of a single test trial is presented in Fig. 2.

Each test trial began with a black fixation cross presented in the centre of the screen for 200 ms. Then, the fixation cross was replaced with a test word (also in black font) for a recognition memory test. At the same time that the test word was displayed, a prompt was presented to remind participants of the recognition instructions and the response options. The prompt “Did you see?” appeared above the test word in red font and the response options (Yes/No) appeared at the bottom of the screen, also in red. The response options were positioned on the screen to correspond to the buttons that would be used to make each response and were counterbalanced across participants. The instruction was to respond “Yes” if the participant recognised the test word from the study phase and to respond “No” if they did not recognise the word, or if they were not sure whether the word had been studied. This display was presented for 3000 ms.

Participants were informed that on each trial on which they recognised the test word, they would be presented with a ‘bonus question’. The “bonus question” was displayed above the test word and the response options were presented below the test word (both in blue font). In the Location Test, the bonus question was either “Was it on the left?” or “Was it on the right?” (counter-balanced across participants). In the Background Test, the bonus question was either “Was it with a Rural Scene?” or “Was it with an Urban Scene?” (also counter-balanced across participants). In each case, participants could respond “Yes”, “No” or “Not sure”. Participants were instructed to make a Yes or a No response only if they could clearly remember the encoding context and were confident in their response. Otherwise, they were instructed to make a “Not Sure” response. The bonus question was displayed for 4000 ms. If participants indicated that they did not recognise the test word, then a fixation cross was displayed until the end of the trial. After this time, a fixation cross was displayed for a 100 ms inter-trial interval.

Finally, a short functional localiser was performed to identify brain regions more responsive to the presentation of scenes than scrambled images. The functional localiser was divided into scene and scrambled-scene blocks. Ten blocks of each type were presented. During each block, twelve scene or scrambled scene images were presented and one image of a smiley face was interspersed in the block at random. Participants were instructed to press any key whenever they saw the smiley face. Each image was shown for 750 ms. Between images, a fixation cross was displayed for 250 ms. In between blocks, there was a 1 s pause before the next block commenced. The entire functional localiser took 6 min to complete.

#### 2.2.3 MRI Acquisition and Analysis

MRI scans were acquired on a Siemens 3-T Prisma scanner equipped with a 32-channel receiver head coil at Great Ormond Street Hospital for Children NHS Foundation Trust, London, UK. BOLD T2*-weighted echo planar functional images were acquired with a flip angle of 75° and a multiband factor of 2. Forty slices, each comprising 2.5 mm isotropic voxels, were acquired with a slice gap of 0.5 mm and a TR of 1240 ms. Over two scanning sessions for the memory test, 1790 functional images were collected, followed by a third session in which an additional 261 images were collected for the functional localiser. T1-weighted anatomical images were acquired with a flip angle of 8°, field of view = 25.6 cm, repetition time = 2300 msec, and 1 mm isotropic voxels.

fMRI pre-processing and analysis were conducted with Statistical Parametric Mapping (SPM8, Wellcome Department of Cognitive Neurology, London, UK), in Matlab R2015a (The Mathworks Inc. USA). Unless otherwise stated, SPM default values were used in all analysis stages. Functional images were subjected to realignment (to the mean image), slice timing correction (using the 17th slice as the reference), reorientation, spatial normalization to a standard EPI template and smoothing with an 8 mm full-width half maximum Gaussian kernel.

The fMRI analysis focuses on scene reinstatement effects (greater activity at test for words that were paired with scenes at encoding relative to words that were paired with scrambled scenes) in the two tasks (Background Task and Location Task). Only test trials correctly endorsed as ‘old’ on the recognition memory test were included in these analyses.

For the Background Task, fMRI analysis was restricted to test trials containing the scene category that was not the subject of the bonus question (referred to as the ‘non-target’ scene category). That is, if the participant received the bonus question “was the word presented with an urban scene?“, then the memory-related activity was examined only for activity elicited either by rural or scrambled scenes. In this way, activity associated with two classes of items (scenes and scrambled scenes) that had the same functional relevance to the memory test could be compared, and, assuming a correct memory judgment, these were both associated with the same “no” response. It is important to note that the scene trials and scrambled scene trials in each task were identical at test (see Fig. 2) and so it is not possible for pre-retrieval processes (e.g. preparation to recall a scene vs. a scrambled scene) to be confounded with retrieval-related scene reinstatement effects in either task.

The fMRI analysis was conducted in two stages, corresponding to subject and group levels. At the subject-level, seven categories of events were modelled with a delta function at each trial onset. In the Background Task, events corresponded to the ‘target’ and ‘non-target’ scene categories, scrambled scenes, and correct rejections of new words (CRs). In the Location Task, trials were modelled as Scene, Scrambled Scene and CRs. Trials associated with false alarms, misses, or a failure to respond, were modelled as events of no interest. The average number of trials for each event of interest was 25.8 for the controls and 19.4 trials for the patients per each event of interest. One patient, DA09, who had a conservative recognition criterion, provided only a few trials for each condition of interest (a minimum of 5 trials). His data are included in the analyses described below; when the analyses were repeated after excluding these data the pattern of significant effects remained the same. The subject level GLMs employed six regressors representing motion-related variance (three for rigid-body translation and three for rotation), as well as regressors modeling the separate scan sessions and the across-scan mean. An AR(1) model was used to estimate and correct for non-sphericity of the error covariance (Friston et al., 2002).

Parameter estimates corresponding to four event categories of interest (scene and scrambled scene trials from each retrieval task) were carried forward to the group level model. These estimates were entered into a mixed-models ANOVA model with factors of task (Background vs. Location), stimulus category (scene, scrambled scene) and Group (Patient vs. Control). Note that SPM employs a single pooled error term in ANOVA models.

For the functional localiser, the onsets of the scene and scrambled scene images were modelled with a delta function convolved with the canonical hemodynamic response function (HRF). The resulting parameter estimates were contrasted at the second level with a mixed-model ANOVA with factors of group (Patient vs. Control) and Stimulus category (Scene vs. Scrambled Scene). As described below, ROIs were defined by reference to voxels that demonstrated a main effect of stimulus category.

The analysis of scene reinstatement was restricted to areas associated *a priori* with scene processing: retrosplenial cortex and parahippocampal cortex. For each region, an ROI was defined by voxels that were more active in the functional localiser during the perception of scenes than scrambled scenes (height threshold of p<0.05, FWE). These functional masks were further restricted by anatomical masks. For the retrosplenial cortex, a mask comprising Brodmann areas 29 and 30 was obtained from the WFU PickAtlas (Maldjian et al., 2003) (http://www.fmri.wfubmc.edu/cms/software). For the parahippocampal cortex, the mask was obtained from the AAL atlas (Tzourio-Mazoyer et al., 2002).

Additional exploratory analyses were also restricted to regions identified by the functional localiser, without the additional constraints of the anatomical masks. In this case, voxel-wise contrasts were height thresholded at p < 0.005 and with a minimum a cluster extent of 45 voxels. The functional localiser mask provided the boundaries for a small volume correction in order to evaluate, for each cluster, whether its peak or its extent survived FWE correction at p < 0.05.

## 3 Results

### 3.1 Behavioural results

Recognition memory performance (Pr) was computed as hit rate minus false alarm rate (Snodgrass and Corwin, 1988). Patients were less able than controls to distinguish old from new words in both the location [t (22) = 5.47, p < 0.001] and background task [(t (22) = 6.44, p < 0.001). Response bias was calculated as Br [(FA/1 – PR); Table 2]. Br did not differ significantly between the two groups.

**Table 2:** Item recognition for patients with DA and controls. Pr is an index of recognition memory performance and Br is an index of response bias.

	Background Task				Location Task			
	Hit	FA	Pr	Br	Hit	FA	Pr	Br
Average Control	0.82	0.10	0.71	0.36	0.84	0.10	0.74	0.38
Average Patient	0.47	0.30	0.17	0.36	0.52	0.24	0.28	0.33
DA01	0.68	0.53	0.14	0.62	0.73	0.32	0.41	0.54
DA09	0.63	0.43	0.19	0.53	0.68	0.50	0.18	0.61
DA12	0.11	0.11	0.00	0.11	0.19	0.08	0.11	0.09
DA15	0.57	0.15	0.42	0.26	0.51	0.18	0.33	0.27
DA16	0.35	0.26	0.09	0.29	0.48	0.10	0.38	0.16

**Fig. 3:**
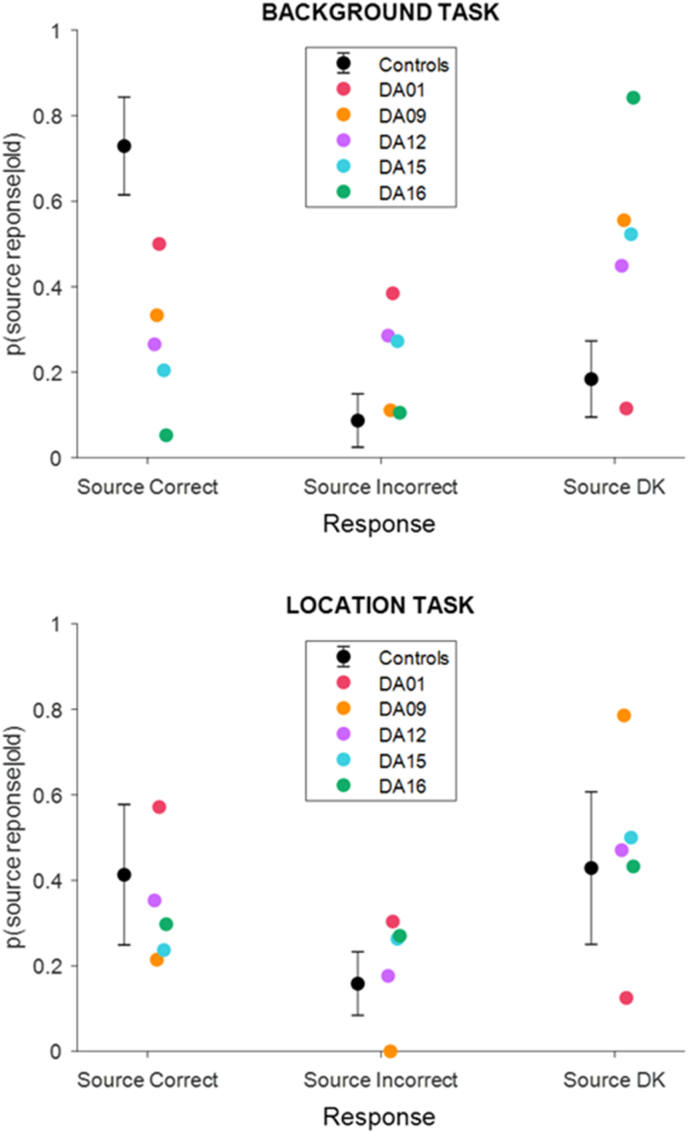
Context memory performance for patients with DA and controls in the Background and Location Tasks (error bar indicates 1 ± standard deviation of the control mean).

Context memory performance is plotted in Fig. 3. Context memory accuracy was estimated as the proportion of correct context judgments out of all correctly recognised “old” words. These data were entered into a mixed-model ANOVA with a within-subject factor of Task (Background Task vs. Location Task) and a between-subject factor of group (Patient vs Control). The ANOVA revealed a significant Task × Group interaction (F (1,21) = 13.0, p < 0.01). When the two tasks were analysed separately, context memory accuracy in the patient group was significantly impaired relative to that of the control group in both the Background Task (t (21) = 7.65, p < 0.001) and the Location Task (t (21) = 2.17, p < 0.05); however, the interaction revealed that the impairment was less pronounced in the latter task.

To evaluate whether memory varied according to the nature of the backgrounds (i.e. rural scenes, urban scenes and scrambled), performance metrics were segregated by background. Thus, Hit Rates, Reaction Times, and Source Memory performance were examined separately according to image type (see Table 3). Source memory was calculated as the probability that an item was associated with a source correct response. The data from control participants were entered into three separate ANOVA models with factors of task (Background Task x Location Task) and encoding condition (Rural Scene, Urban Scene, Scrambled Scene). Mauchly's Test of Sphericity indicated that the assumption of sphericity had not been violated. For each analysis, no significant effects of encoding condition were revealed. There was no effect of encoding condition on the item hit rate F (2,36) = 2.80, n.s., nor was there an interaction between task and encoding condition on item hit rate F (2,36) = 1.12, n.s. Likewise, there was no effect of encoding condition on reaction times F (2,36) = 2.83, n.s., nor an interaction between task and encoding condition on reaction times F (2, 36) = 0.10, n,s. Finally, there was no effect of encoding condition on source memory performance, F (2, 36) = 1.03, n,s. nor an interaction between task and encoding condition on source memory, F (2,36) = 0.02, n.s. These data indicate that fMRI effects are unlikely to be driven by differences in the memory strength of items encoded alongside scenes compared to scrambled scenes.

**Table 3:** Item recognition for patients with DA and controls. Pr is an index of recognition memory performance and Br is an index of response bias. Source memory is calculated as the probability that a test item is associated with a correct source response.

	Controls		Patients with DA	
Test Phase	Location Task	Background Task	Location Task	Background Task
Item Hit Rate				
Rural Scene	0.86 (0.11)	0.82 (0.17)	0.58 (0.19)	0.48 (0.23)
Urban Scene	0.85 (0.11)	0.86 (0.14)	0.48 (0.20)	0.42 (0.24)
Scrambled Scene	0.81 (0.16)	0.78 (0.13)	0.51 (0.22)	0.50 (0.19)
Test RT (ms)				
Rural Scene	1564 (222)	1556 (190)	1885 (237)	1886 (227)
Urban Scene	1526 (213)	1516 (197)	1842 (238)	1940 (259)
Scrambled Scene	1573 (165)	1571 (235)	1804 (179)	1782 (204)
Source Memory				
Rural Scene	0.46 (0.23)	0.72 (0.16)	0.38 (0.04)	0.25 (0.19)
Urban Scene	0.37 (0.17)	0.73 (0.15)	0.22 (0.24)	0.22 (0.27)
Scrambled Scene	0.40 (0.17)	0.74 (0.12)	0.37 (0.18)	0.25 (0.24)

### 3.2 ROI analysis

**Fig. 4:**
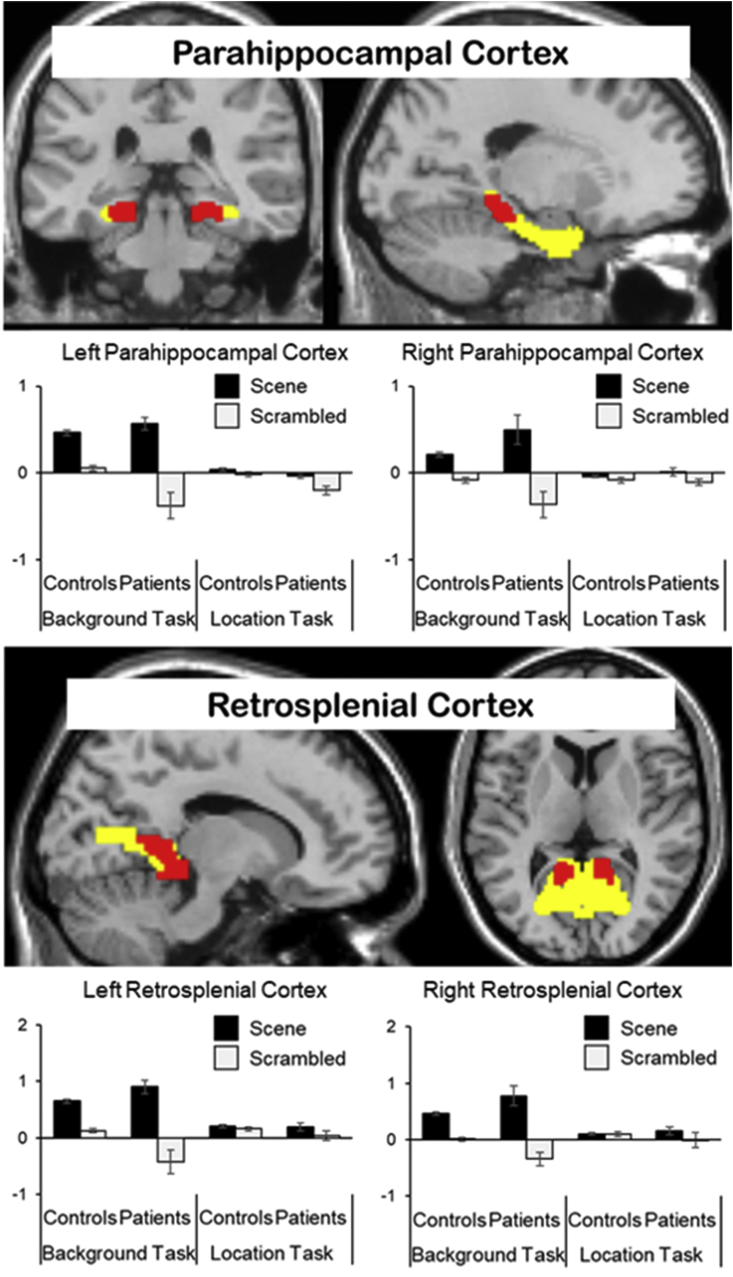
ROIs (shown in red) were defined by the functional localiser (greater activity to scene stimuli than scrambled scene stimuli, FWE, p < 0.05) and were further constrained by an anatomical mask of the region of interest (shown in yellow). Parameter estimates (arbitrary units) plotted in bar charts were extracted from these ROIs during the memory retrieval tasks. (For interpretation of the references to colour in this figure legend, the reader is referred to the Web version of this article.)

The ROIs for the retrosplenial cortex and parahippocampal cortex are plotted in Fig. 4 (see MRI Acquisition and Analysis for a description of their derivation). Mean voxel-wise parameter estimates were extracted from each ROI and entered into a 2x2x2x2 mixed models ANOVA with factors of Hemisphere (left vs right), Task (Background vs. Location), study background (words that had been previously encoded in association with a scene versus a scrambled scene), and Group (Patients with DA vs. controls). All significant effects that included an interaction with the factor of study background are described.

In the parahippocampal cortex, there was a significant main effect of study background, indicating that scene reinstatement effects could be identified in this region, F (1, 21) = 13.7, p < 0.001. In addition, there was a significant interaction between study background and task F (1, 21) = 8.19, p < 0.01, indicating that reinstatement effects were moderated by retrieval task see Fig. 4). The three-way interaction between task, study background and group was not significant F (1,21) = 2.37), n.s. There was, however, a significant interaction between group and study background, which was driven by larger reinstatement effects for patients than for controls, F (1, 21) = 4.64, p < 0.05.

The findings for the retrosplenial cortex were similar to those reported above for the parahippocampal cortex. There was a significant main effect of study background, indicative of scene reinstatement, F (1, 21) = 9.65, p < 0.01. These reinstatement effects were moderated by task F (1,21) = 9.29, p < 0.01 in the absence of a three-way interaction between F (1, 21) = 2.01, n.s. There was a non-significant trend for reinstatement effects to be larger in patients than controls, F (1, 21) = 2.44, p = 0.07.

**Fig. 5:**
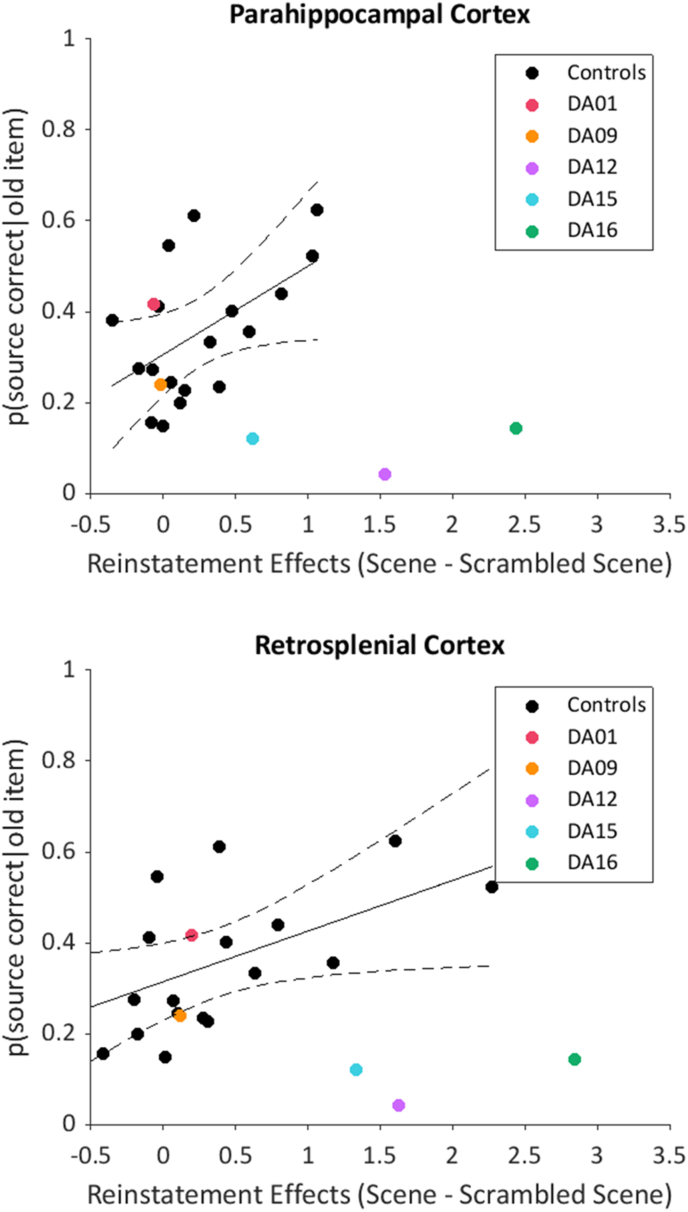
Correlation between reinstatement effects in the background task and memory performance in controls. Dashed lines represent 95% confidence intervals. Patient data are plotted for illustrative purposes only. Note that DA01 made a source correct response on 40% of the trials, however, this patient also made a source incorrect response on a similar proportion of trials and rarely used the “don't know” response. Thus, although this patient appears to be performing as well as controls, this was entirely due to a liberal response bias. (see context memory data, Fig. 3).

Reinstatement effects during the background task were computed for each ROI (scene – scrambled scene) and correlated with context memory performance in the same task. This correlation was computed for controls only. The correlation in the parahippocampal ROI was significant (Pearson's correlation coefficient [N = 18] = 0.53, p < 0.05; two-tailed) as was the correlation in the retrosplenial cortex (Pearson's correlation coefficient [N = 18] = 0.54, p < 0.05; two-tailed) suggesting that larger reinstatement effects were associated with better memory for the scene stimuli in controls. These data are plotted in Fig. 5.

### 3.3 Whole brain analysis

In addition to the hypothesis-driven analysis above, we conducted an exploratory analysis of scene reinstatement effects in patients with DA. The purpose of this analysis was to identify regions where patients and controls demonstrated cortical reinstatement effects, and to identify regions where the effects diverged between the groups.

**Fig. 6:**
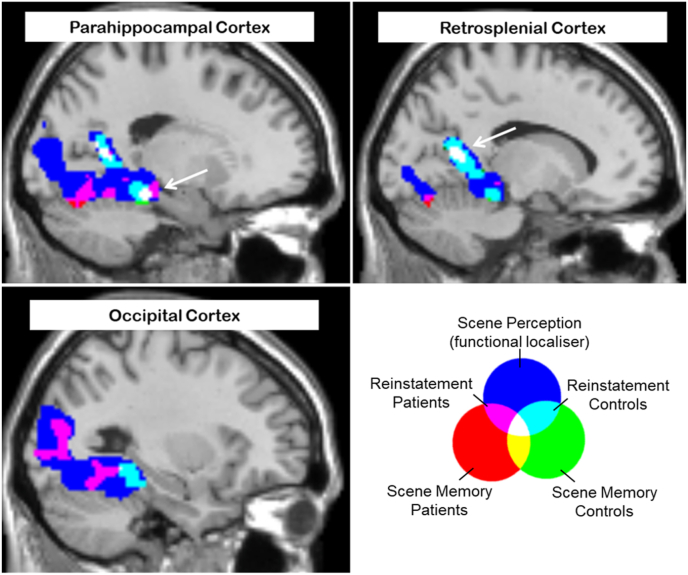
Memory effects in patients (red) and controls (green) represent regions that are more active during the memory test for words that were previously paired with a scene than a scrambled scene. Perception effects represent regions that are more active during the perception of scenes than scrambled scenes during the functional localiser. All contrasts are shown with a threshold of 0.005 and a cluster extent of 45 voxels. (For interpretation of the references to colour in this figure legend, the reader is referred to the Web version of this article.)

In pursuit of these aims we employed an inclusive masking procedure to identify voxels that were more active for scene stimuli than for scrambled scene stimuli in the functional localiser (scene perception effects) and were more active for test items in the Background Task that were associated with scenes than scrambled scenes (scene memory effects). This procedure was performed separately for the patient and control groups. In each case, the scene memory effects and the scene perception effects from the functional localiser were entered at p < 0.005 with a cluster extent threshold of 45 voxels. The results of this analysis are shown in Table 4 and Fig. 6. Patients and controls both demonstrated scene reinstatement effects in the parahippocampal and retrosplenial cortex (replicating the results of the ROI analysis), but interestingly, the patients also demonstrated reinstatement effects extending posteriorly towards the occipital cortex, these effects were not evident in controls.

**Table 4:** Regions showing scene reinstatement effects in patients and controls P < 0.005, 45 voxels. FWE corrected cluster-wise and FWE corrected peak refer to the outcomes of a small volume correction with the functional localiser mask.

Group	Region	x	y	z	Cluster Size	Peak Z
Patients	Left Thalamus**	−8	−32	−2	51	3.84
	Left Parahippocampal Gyrus	−18	−30	−17	–	3.09
	RightCerebellum/Fusiform Gyrus*	30	−55	−2	207	3.72
	Right Thalamus	15	−30	1	74	3.71
	Left Cerebellum/Fusiform Gyrus*	−16	−60	−14	379	3.70
	Left Occipital Gyrus	−33	−90	1	205	3.63
	Right Middle Occipital Gyrus*	42	−75	25	253	3.39
Controls	Right Retrosplenial Cortex**	17	−52	10	192	4.04
	Left Retrosplenial Cortex**	−16	−47	4	138	3.93
	Left Parahippocampal Gyrus	−23	−32	−17	106	4.04

*p < 0.05 FWE corrected cluster-wise.

**p < 0.05 FWE corrected peak.

Although these are exploratory analyses, a small volume correction was conducted to provide a principled correction for multiple comparisons. The search space was restricted to voxels identified in the functional localiser (Scene > Scrambled Scene, p < 0.005, 45vox). Within this search volume, the set-level (likelihood of obtaining the observed number of clusters by chance) of significance across the three clusters identified in controls was significant (p < 0.05) and the set-level significance across the six clusters identified in patients was also significant (P < 0.001). The family wise error corrected p values are presented in Table 4.

**Fig. 7:**
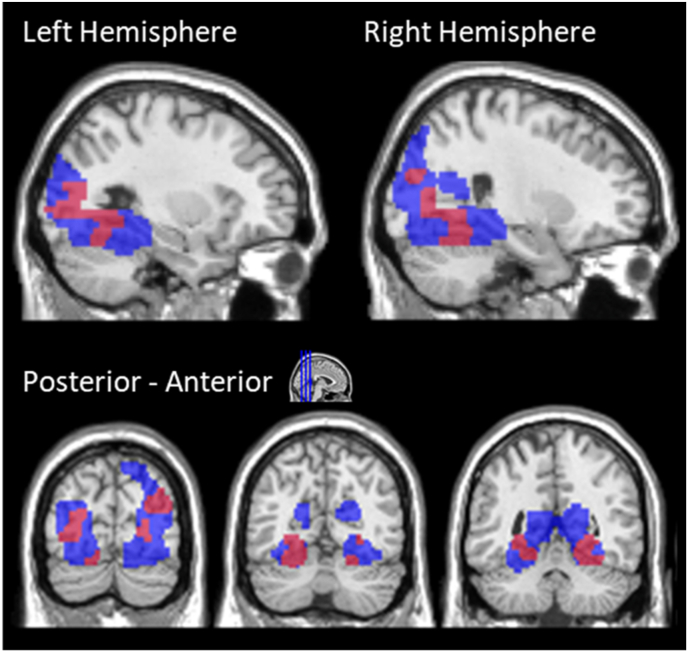
Regions where reinstatement effects in the Background Task were greater in the DA patients than the controls (p < 0.005, 45 voxels) shown in red, after inclusive masking with the functional localiser (p < 0.005, 45 voxels) shown in blue. Fig. 7: Regions where reinstatement effects in the Background Task were greater in the DA patients than the controls (p < 0.005, 45 voxels) shown in red, after inclusive masking with the functional localiser (p < 0.005, 45 voxels) shown in blue. (For interpretation of the references to colour in this figure legend, the reader is referred to the Web version of this article.)

Finally, we were interested in whether the patients showed significant reinstatement in any regions where controls did not show reinstatement. We performed a directional interaction contrast (thresholded at p<0.005 and 45 voxels) to identify regions where scene memory effects in the Background Task were greater in patients than in the controls. This analysis was restricted to the functional localiser mask (p < 0.005, 45 vox). This analysis revealed four clusters (reported in Table 5) that extended posteriorly from the fusiform to the occipital cortex bilaterally (see Fig. 7 and Table 5). Although these are exploratory analyses, a small volume correction was conducted to provide a principled correction for multiple comparisons. The search space was restricted to voxels identified in the functional localiser (Scene > Scrambled Scene, p < 0.005, 45vox). Within this search volume, the set-level of significance across the four clusters was significant (p < 0.001).

**Table 5:** Regions where reinstatement effects were larger in patients than in controls.

Region	Whole-Brain Analysis				
	x	y	z	Cluster Size	Peak Z
Left Fusiform Gyrus*	−13	−77	−20	437	3.61
Left Inferior Occipital	−33	−90	1	–	3.85
Right Fusiform Gyrus	30	−55	−2	164	3.64
Right Lingual Gyrus	27	−72	4	50	3.25
Right Middle Occipital Gyrus	42	−75	25	129	3.13

*p < 0.05 FWE corrected cluster-wise.

The reverse contrast showed no regions in which reinstatement effects were larger for controls than patients with DA.

## 4 Discussion

The primary aims of this study were to examine whether patients with DA show cortical reinstatement effects and, if so, whether these effects are sensitive to retrieval goals. Below, we discuss these issues in turn, and then discuss how the results enhance the understanding of DA as a memory disorder.

### 4.1 Cortical reinstatement and episodic memory

We used cortical reinstatement as an objective method for the assessment of memory retrieval in patients with DA. Remarkably, our sample of patients demonstrated scene reinstatement effects that overlapped with those identified in healthy controls. Despite the presence of these effects, the patients were severely impaired in their ability to access the reinstated scene information to support their memory responses. This dissociation suggests that the associated ‘context’ of a prior event may be represented in the cortex, yet explicit, ‘declarative’ memory for the same event may fail.

Importantly, these fMRI reinstatement effects cannot be explained by pre-retrieval processes, such as those associated with “retrieval orientation”. In the Background Task, the images paired with the test words were relevant to the retrieval goal and so participants may have adopted a retrieval orientation to facilitate retrieval of these images (see Strategic Retrieval). Crucially, however, the test trials were identical within each task (see Fig. 2). Notably neither the task cue nor the “bonus question” informed participants which type of image (e.g. a rural scene, a urban scene, or a scrambled scene) to prepare for, and so participants were not able to differentially adopt image-specific preparatory sets in response to the test instructions. Nonetheless, there was greater BOLD signal for studied words paired with scenes rather than scrambled scenes in cortical regions selectively associated with scene processing. The neural activity underlying this increased signal must, therefore, reflect retrieval-related reinstatement of scene information that had been encoded during the study phase. This leads to the seemingly counter-intuitive conclusion that patients with DA can implicitly retrieve specific contextual features associated with a study event and reinstate these features in the cortex.

Although counter-intuitive, this finding is consistent with data acquired in healthy controls. Thakral et al. (2015) reported that typical young adults demonstrated equivalent retrieval-related reinstatement effects (as operationalized by the accuracy of a MVPA classifier) for events that they could recollect and events that were endorsed as familiar only. In addition, under stressful circumstances, healthy participants were reported to be less able to accurately remember details of a prior experience than in a low stress condition, despite showing equivalent reinstatement effects (Gagnon et al., 2018). These findings from healthy participants, together with the data reported here from our amnesic patients, suggest that a prior event can be cortically reinstated yet remain inaccessible to conscious report. Successful recollection must, therefore, depend on *more* than the reactivation of the cortical activity elicited by the prior event as it was experienced, although it is far from clear what additional processing might be necessary to enable recollection (Thakral et al., 2017). Perhaps additional hippocampal-dependent interaction with the cortical reinstatement is necessary for the experience of recollection.

Another possibility is that some contextual information about the prior episode was recovered, but the retrieved memories lacked the detail necessary to support the required urban/rural scene judgement. Perhaps if we had asked patients a more general question, such as whether the test word had been paired with a scene or a scrambled scene, they might have shown performance more congruent with their scene reinstatement effects. To address this question, we asked DA01 to undertake a second memory test in which the encoding phase was identical to that employed in the fMRI study described here, but the retrieval task required him to indicate merely whether recognised words had been presented against a scene (rural or urban) or a scrambled image. Reminiscent of his performance in the present study, DA01 performed at chance. This finding strongly suggests that the neural reinstatement effects observed in DA patients are not sufficient to support accurate recollection even of highly generalized (gist-like) contextual information.

Perhaps surprisingly, our exploratory whole-brain analyses identified areas where scene reinstatement effects were stronger in patients than controls. We did not anticipate such a finding, and it should be regarded as preliminary. Notably, most of these effects did not survive FWE small volume correction. One interpretation of these effects, should they prove reproduceable, is that they reflect functional re-organisation of the cortex in the patients with DA in compensation for their early hippocampal damage. There is an emerging literature that links the visual cortex with learning and memory in humans and rodents (Cooke and Bear, 2015; Rosen et al., 2018; Rosenthal et al., 2016). For example, Rosenthal et al. (2016, 2018) reported that primary visual cortex is sensitive to associative memory for complex visual sequences. One possibility is that the low-level representational capabilities of sensory systems are recruited in DA to support associative memory. Such reorganisation however does not lead to improved episodic memory performance. Further research will be necessary to establish whether patients with DA recruit visual cortex to support associative memory in the presence of hippocampal pathology (e.g. by supporting visual associative recognition memory in the absence of recollection, or supporting semantic memory, see Implications for Semantic Learning).

Finally, it is worth discussing the relevance of these data to our understanding of DA. The present findings suggest that while patients with DA do not consciously recall episodes from their past, the neural representation of reactivated episodic information is remarkably similar to that of healthy controls. Our findings suggest that episodic (i.e. trial unique) information, including the binding of an item with its context, can be successfully encoded. This bound episodic memory trace is stored for some time, and then *reactivated* during a memory test to the extent that it is later recapitulated in the cortex (although remaining inaccessible to recall). Thus, the stumbling block in these patients might not encompass the entire memory stream (i.e. encoding – storage – retrieval), but rather, is specific to enabling the access of reactivated mnemonic information to processes that control memory-guided behaviour. There is some prior evidence in support of this conjecture from the report of patient Neil (Vargha-Khadem et al., 1994), who had a dense episodic amnesia inasmuch as he was unable to recall everyday events, but had the remarkable ability to retrieve post morbid memories through the act of writing, without having any awareness, at least to oral report, of the content of his written report. More recently (St-Laurent et al., 2020), described a patient with developmental amnesia (as a consequence of a thalamic stroke in infancy) who nonetheless showed fMRI evidence of content-specific retrieval of memories of short video clips. Taken together, the present and these prior findings point to an emerging literature suggesting that some degree of episodic memory retrieval, at least at the neural level, may occur in patients with DA.

### 4.2 Strategic retrieval

We were interested in the question of whether patients with DA would show evidence of strategic retrieval processing. Our previous work indicated that healthy adults demonstrated attenuated scene reinstatement effects in the parahippocampal cortex when scene memory was irrelevant to the retrieval goal, but this finding did not extend to retrosplenial cortex. We interpreted this regional dissociation as evidence that strategic control processes were engaged to dampen reinstatement of fine-grained scene information in the parahippocampal cortex, while allowing the “gist” of the background image to be reinstated in the retrosplenial cortex (Elward and Rugg, 2015). In the present study, however, attenuated scene reinstatement effects in the location task were evident in both cortical regions. Indeed, there was no detectable evidence of reinstatement in either region during the location task.

The disparity between the prior and present findings may reflect the differing designs of the two studies. Unlike in the earlier study, here participants were not required to switch unpredictably between the two tasks on a trial-by-trial basis. Rather, the task manipulation was blocked, such that the background images maintained their irrelevance over successive trials of the location task. This may have enabled the deployment of more effective strategic control operations. Similarly, Srokova et al. (2020) found that young adults showed attenuated scene reinstatement effects in both the retrosplenial cortex and the parahippocampal cortex using a blocked paradigm (Srokova et al., 2020). Regardless of this issue, together with our previous report (Elward and Rugg, 2015), the present findings suggest that reinstatement of a prior event is not “all-or-none” (Norman and O'Reilly, 2003), but rather, a controlled process that can be strategically aligned with retrieval goals. Remarkably, this process appears to be intact in patients with DA. Thus, at least some strategic mnemonic processes appear to develop normally in the absence of a normally functioning episodic memory system.

### 4.3 Behavioural performance

Patients with DA have been characterized as having preserved recognition memory (Adlam et al., 2009; Patai et al., 2015). Consistent with this characterization, our patients performed within the normal range on standardized tests of recognition (see Table 2). However, all of the patients were less able to recognise words from the encoding phase than controls. This finding is reminiscent of studies with patients who sustained hippocampal damage in adulthood; such patients have difficulty with semantic memory and recognition memory after injury (Jeneson et al., 2010; Kirwan et al., 2010), whereas patients with DA typically show preserved (or even superior) semantic memory and recognition memory abilities (Adlam et al., 2009; Jonin et al., 2018; Patai et al., 2015). A likely explanation for the present finding is that recognition memory in our experimental procedure depended more heavily on recollection (and less on familiarity) than do the recognition memory tests typically used to assess patients with DA. In our paradigm, participants were presented with 180 to-be-remembered words and images and the test words were presented in a different font colour from that employed at encoding, (i.e. they were not exact “copy cues”). Together, these factors may have limited the utility of familiarity-based judgements. In such circumstances, a recognition impairment in the patient group would become apparent. Control participants, who have access to normally-functioning recollection to support their responses, are therefore at a considerable advantage. Thus, to the degree that item recognition in the present study was dependent on recollection, it would be expected to be impaired in the patient group.

### 4.4 Implications for Semantic Learning

In the introduction we noted that patients with DA are able to develop good semantic memory, that is, memory for information that generalises across multiple events, in spite of an inability to recall the prior events of their lives in which this semantic information was encountered. We noted a range of theoretical positions about the role of the hippocampus in supporting semantic memory in typically-developing children and adults. Mishkin et al. (1997) posited that in DA semantic memory may be supported by the perirhinal and entorhinal cortices independently of hippocampal-dependent episodic memory. More recently, Miyashita (2019) suggested that the perirhinal cortex in particular can support memory for associative relations in both humans and non-human primates in service of semantic-like memory. In an exceptionally large cohort of patients with DA, it was repoted that perirhinal, entorhinal and parahippocampal cortices were not significantly reduced in volume compared to a well-matched group of controls (Chareyron et al., 2021); therefore, these cortices may support semantic learning in patients with DA. Thus, semantic memory may have developed in patients with DA because of an implicit cortical memory mechanism. This mechanism develops early in life and enables infants and young children to learn language, concepts and other semantic information without autonoesis. In typically-developing children, a second hippocampal-dependent memory process emerges in later childhood that enables past events to be re-experienced in autonoetic consciousness and recalled. In patients with DA, however, this second memory process does not emerge effectively because of their severe hippocampal damage (Chareyron et al., 2021). Consequently, those affected have lifelong difficulty with autonoetic consciousness and recall of their personal experiences. Implicit memory processes, however, continue to operate throughout life in patients with DA. The present data indicate that cortical reinstatement may be one such implicit process that can allow for the associated details of a prior event to be made available for cortical learning, regardless of whether the details are accessible to recall. In future work, if we can establish that learning experiences that are explicitly ‘forgotten’ are nonetheless available to cortical reinstatement, and that this reinstatement is an important determinant of subsequent semantic memory ability, this will shed light on how those who cannot recall are nonetheless able to learn from their life experiences.

## Credit author statement

Rachael Elward: Conceptualization, Methodology, data collection (Investigation, administration), Data curation, Formal analysis, Visualization, Writing – original draft Writing – original draft. Writing – review & editing, Michael Rugg: Methodology, Formal analysis, Writing – review & editing. Faraneh Vargha Khadem: Conceptualization, Funding acquisition, Investigation, Project administration, Resources, Supervision, Writing – review & editing.
